# Benchmarking Functionals for Strong-Field Light-Matter Interactions in Adiabatic Time-Dependent Density Functional Theory

**DOI:** 10.1021/acs.jpclett.4c01383

**Published:** 2024-07-08

**Authors:** Ofer Neufeld, Nicolas Tancogne-Dejean, Angel Rubio

**Affiliations:** † Max Planck Institute for the Structure and Dynamics of Matter and Center for Free-Electron Laser Science, Hamburg 22761, Germany; ‡ Center for Computational Quantum Physics (CCQ), The Flatiron Institute, New York, New York 10010, United States

## Abstract

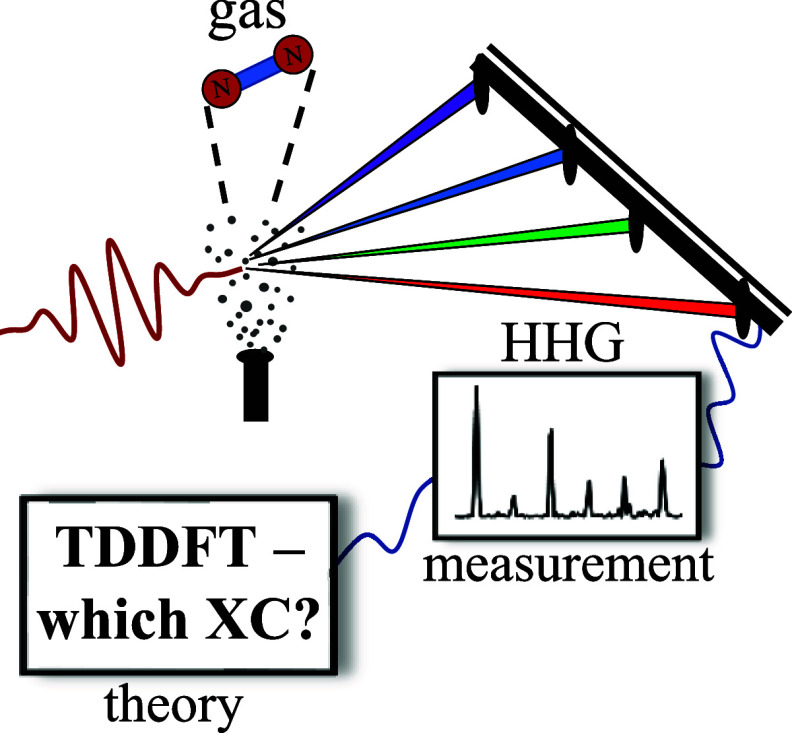

In recent years, time-dependent density functional theory (TDDFT) has been extensively employed for highly nonlinear optics in molecules and solids, including high harmonic generation (HHG), photoemission, and more. TDDFT exhibits a relatively low numerical cost while still describing both light-matter and electron–electron interactions ab initio, making it highly appealing. However, the majority of implementations of the theory utilize the simplest possible approximations for the exchange-correlation (XC) functional–either the local density or generalized gradient approximations, which are traditionally considered to have rather poor chemical accuracy. We present the first systematic study of the XC functional effect on molecular HHG, testing various levels of theory. Our numerical results suggest justification for using simpler approximations for the XC functional, showing that hybrid and meta functionals (as well as Hartree–Fock) can, at times, lead to poor and unphysical results. The specific source of the failure in more elaborate functionals should be topic of future work, but we hypothesize that its origin might be connected to the adiabatic approximation of TDDFT.

High harmonic generation (HHG) is a highly nonlinear optical process that occurs when gas, liquid, or solid media are irradiated by intense lasers.^1−8^ From the optical point of view, HHG is a frequency upconversion process, whereby multiple pump laser photons annihilate and emit a higher energy photon. From the point of view of the material medium, HHG occurs as a result of the classical laser field driving rapid charge motion within, and outside, of the sample.^3,8−16^ The motion of electrons on attosecond and sub-laser-cycle timescales acts as an instigator that allows for charge recombination and high-frequency light emission. In that respect, to theoretically describe the microscopic physics responsible for HHG, a quantum mechanical theory that captures the interactions of charges inside the sample with the externally applied laser, as well as with each other, is required.

Historically, HHG has been predominantly analyzed with noninteracting models (employing the independent particle approximation (IPA)).^2,9,10,13,17−38^ Moreover, due to the energy level landscape in atomic systems where HHG was pioneered,^39^ a single active electron (SAE) approximation is typically employed, where contributions to HHG from any orbitals, except the topmost, are neglected. In this framework, one can also neglect long-range Coulombic effects under the strong-field approximation (SFA),^40^ providing an intuitive semiclassical picture for the HHG mechanism. Such models (ranging from IPA to SFA) usually perform very well, correctly capturing HHG cutoff energies, plateau structure, time-frequency, and polarization properties of the emitted harmonics, permitting various ultrafast spectroscopies. While there has been much interest over the years in the role of correlations and multielectron effects in HHG, these usually contribute negligibly to the nonlinear response unless on resonance,^41−54^ in strongly correlated systems,^55−59^ or under special circumstances.^54,60−63^ Nevertheless, it is difficult to predict a priori if they should be included, and it is generally unknown if, under certain conditions, correlations can be induced by interactions with intense lasers. To overcome this, two competing approaches are employed: (i) improving simpler models by incorporating additional interactions (e.g., corrected SFA^64^), (ii) using ab initio techniques without a priori assumptions (e.g., TDDFT,^65^ multiconfiguration methods,^53,66−68^ R-matrix,^69,70^ etc.).

Among the ab initio methodologies, the most common theory level is time-dependent density functional theory (TDDFT) employing the adiabatic approximation for the exchange-correlation (XC) functional.^65,71^ TDDFT offers a substantially reduced numerical cost compared to wave function methods, while still accurately capturing interactions of electrons with nuclei, the laser, and each other. It is especially appealing for calculations involving larger molecules, solids, or longer laser pulse setups, where wave function methods are impractical with current technology. At the same time, the theoretical foundation of employing TDDFT in this manner remains vague, because (i) under such intense laser irradiation the adiabatic approximation might break down due to the very fast changes to the electron density^71−75^ (although it remains an open problem to identify whether it indeed breaks down, and in certain systems this was shown not to be the case^76,77^), and (ii) it is unknown which XC functional is better suited for describing HHG, since functional benchmarking has not systematically been done for highly nonlinear optical phenomena, unlike in ground-state calculations. Indeed, usually the adiabatic local density (LDA) or generalized gradient approximations (GGA) are employed without justification and due to numerical simplicity.^78^ This issue is further complicated by the fact that comparisons between theory and experiment are in any case not quantitative due to many factors that affect HHG but are not described in theory, such as macroscopic physics (e.g., phase matching, reabsorption, focal averaging, etc.),^32^ vibrational modes,^79−81^ temperature,^82,83^ and sometimes even simple experimental uncertainty of the laser pulse parameters. Up to this point, TDDFT was only validated against wave function-based methods in a handful of simple oriented molecules.^84−86^ However, such calculations are not necessarily even a proper benchmark due to approximations employed in the wave function methodology.

Another approach is to select qualitative yet distinct spectral features in measurements and use those for benchmarking theory. The most common occurrences are resonance emission enhancement,^44,87^ or spectral minima.^88,89^ However, here several levels of theory could agree all the way from IPA to fully correlated methods. In certain cases, HHG minima might even be better reproduced by TDDFT at the LDA level rather than configuration interaction (CI) calculations, as was recently shown for nitrogen.^84,90,91^ This approach is therefore potentially unsatisfactory as criteria for theory level section.

Nevertheless, due to over three decades of theoretical and experimental research in gas-phase HHG, we argue here that some parts of the microscopic response can be straightforwardly benchmarked without directly comparing to experiments. In particular, the cutoff dependence on the system and laser parameters and the typical time-frequency characteristics of HHG should follow an expected shape that has been consistently measured in multiple systems and conditions over the years. In that respect, a given HHG calculation should uphold certain physical expectations, regardless of which level of theory is employed. This includes maximal obtained electronic kinetic energies, timings for energy-resolved emission, structural signatures, and more. Using such features, it might be possible to access (even partially) the physical validity of different functionals.

Here we follow this logic and compare XC functionals for adiabatic TDDFT in the context of highly nonlinear optics from molecular gases. We focus on commonly employed laser conditions and chemical systems: nitrogen and water. We test six different XC functionals along Jacob’s ladder,^92^ starting from the LDA, GGA (using Perdew–Burke–Ernzerhof (PBE) XC),^93^ meta-GGA (mGGA) (using the local Minoseota-2015 (MN-15L) XC that was shown to have quite broad chemical accuracy,^94^ and the re-regularized version^95,96^ of the strongly constrained and appropriately normed (SCAN) XC^97^), and hybrids (using B3LYP^98^ that is very common in chemistry, and the nonempirical widely applicable PBE0^99^). We further test standard time-dependent Hartree–Fock theory (TDHF), as well as the inclusion or exclusion of self-interaction corrections (SIC).^100^ From this general analysis, we conclude the following: (i) Simpler GGA functionals perform very well and provide clean HHG spectra with the expected cutoff positions and time-frequency characteristics. (ii) The application of a SIC drastically improves the results with almost no additional cost. (iii) mGGA functionals can predict unphysical time-frequency characteristics in the plateau region and a much-higher-than-expected cutoff energy. (iv) Hybrid functionals (as well as TDHF) might produce equivalent results to GGA in some systems and laser conditions (without any apparent added chemical accuracy), but might also produce too-high cutoffs and unreasonable time-frequency characteristics. Our work therefore provides justification for the application of LDA and GGA in TDDFT for highly nonlinear optics, and should stimulate further research for understanding the failures of meta and hybrid functionals.

We begin by describing our examined systems and methodological approach. For nitrogen (N_2_) and water (H_2_O), we perform ground-state (GS) DFT calculations to obtain the initial Kohn–Sham (KS) orbitals at *t* = 0. All calculations were performed using Octopus code^101−104^ on a real-space grid representation and without assumed symmetries. We neglected spin degrees of freedom and spin–orbit coupling. We solved the time-dependent KS equations, given in atomic units and the length gauge by
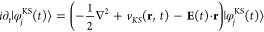
1where |φ_*j*_^KS^(*t*)⟩ is the *j*th time-dependent KS orbital and *v*_KS_(**r**,*t*) is the time-dependent KS potential:
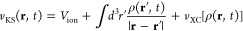
2where *V*_ion_ represents interactions of electrons with nuclei and core electrons (within the frozen core approximation and employing pseudopotentials^105^), ρ(**r**,*t*) is the time-dependent electron density, and *v*_XC_ denotes the XC functional in the adiabatic approximation (which is generally also orbital-dependent). Note that we employ Dirac notations throughout for orbitals while keeping explicit spatial dependencies for operators whenever possible. **E**(*t*) in eq 1 is the time-dependent electric field of the driving laser pulse, which in the dipole approximation was taken as
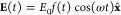
3where *E*_0_ is the field amplitude (connected to the peak power *I*_0_), *f*(*t*) is a temporal envelope, and ω is the carrier frequency. We employed the frozen nuclei approximation (neglecting ion motion) and a complex absorbing potential (CAP) during propagation to avoid unphysical reflections of electrons from the boundaries. The total response was orientation-averaged using trapezoidal weights on an Euler grid with a method similar to refs (106) and (107). From the time-dependent density, we obtained the dipolar response:
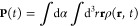
4with the α integral representing angular averaging. From **P**(*t*), we obtained the HHG spectral power:
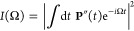
5where **P**^′′^(*t*) is the second time-derivative of **P**(*t*), denoting the dipole acceleration in the medium, and the integral is evaluated with a fast Fourier transform. For TDHF, we solved the corresponding time-dependent Schrodinger equation with the Fock operator replacing the Hartree and XC part of the TDDFT equations of motion. All other technical details about the grid, molecular geometries, propagation scheme, etc., are delegated to the Supporting Information (SI).

Our main physical question of interest is what effect does the choice of XC functional and theory level have on emerging HHG spectra? We first address this question in N_2_, which is a high-symmetry (*D*_∞_) well-studied system in HHG.^108−111^ In particular, N_2_ exhibits a well-known structural minimum at ∼39 eV. This feature was experimentally shown to be very robust under laser wavelength and intensity variations, as well as under alignment.^89,111^ It has been reproduced by TDDFT quite well at the level of LDA and GGA, but has recently been shown to be overshot in CI calculations.^84,90,91^ Our initial approach is to test which level of theory captures this feature. Unfortunately (or fortunately), all tested functionals produce a sharp minima at essentially the exact and expected position of ∼39 eV (see Figure 1 and red arrows). This includes LDA and PBE functionals (with and without a SIC), B3LYP, PBE0, TDMN-15L, and r^2^SCAN. The only method that completely fails in producing any spectral minima is TDHF. The failure of TDHF is very surprising, given that the feature arises from the molecular geometry and orbital nature itself. In this context, note that the HF produces different level alignment, compared to other methods, where the highest-occupied molecular orbital (HOMO) and HOMO–1 N_2_ KS-MOs are degenerate instead of the HOMO–1 and HOMO–2 MOs (see the SI).^112^ Since the minima arises from interferences, this could potentially be the cause of the minima not being reproduced, hinting at a failure of HF at the ground state level rather than a dynamical one.

**Figure 1:**
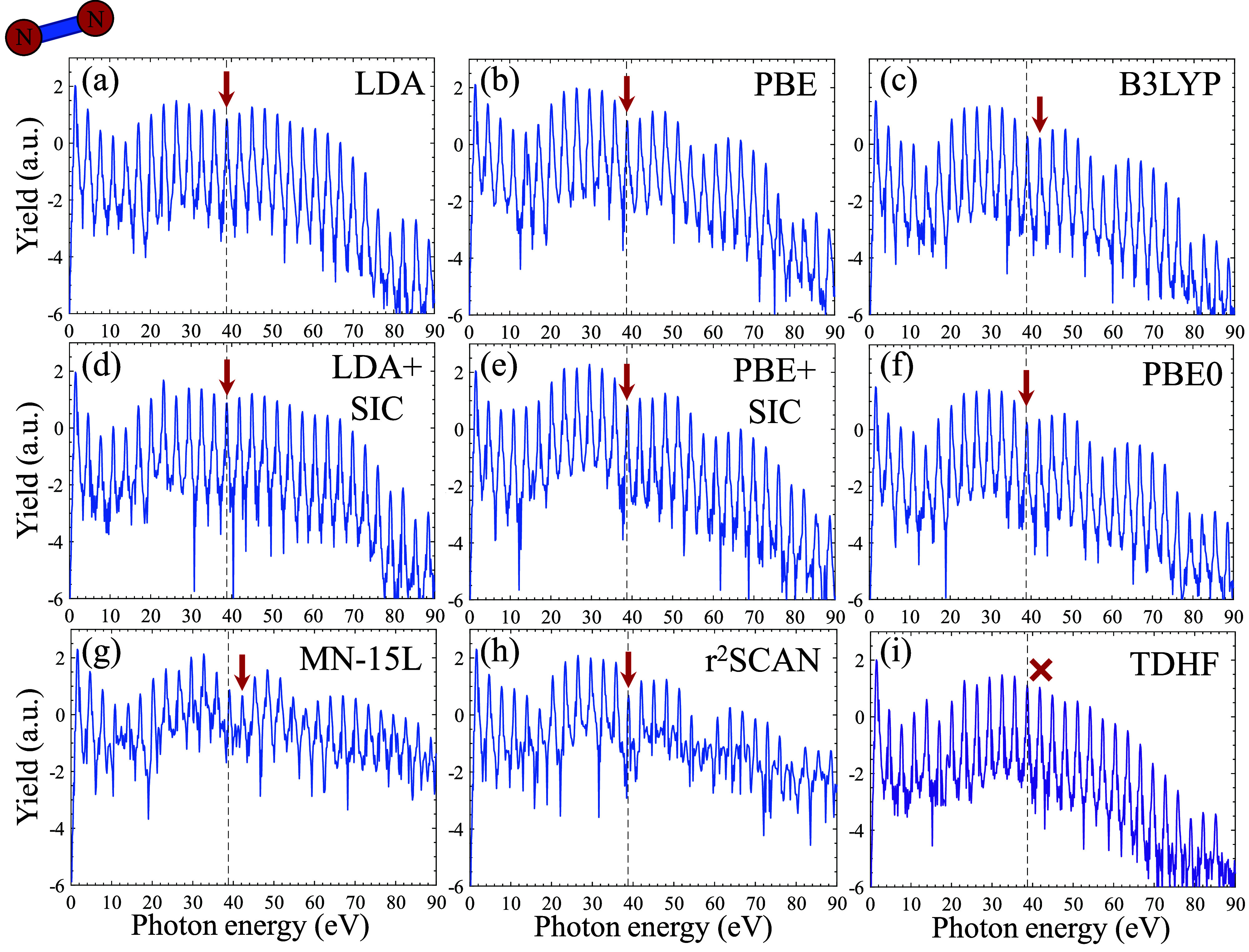
HHG spectra from aligned N_2_ driven by 800 nm pulses at a power of 2 × 10^14^ W/cm^2^ with different levels of theory. Red arrows indicate the position of a structural minima in the calculations, and black dashed lines indicates the experimentally measured position of the minima. Plot is given in logarithmic scale.

Following this failed attempt to isolate an ideal functional choice for HHG, we move on to explore other spectral characteristics. Note that, already at the ground-state level, each choice of functional changes the ionization potential (*I*_*p*_) of the system and the relative KS eigenenergies. Deeper eigenenergies do not correspond to ionization energies in TDDFT (which can be altered by interactions); however, in practice, their difference is small enough that HHG,^113^ which is believed to be relatively insensitive to electron interaction, can be analyzed taking them to be equal. We further note that the type of SIC that we employ does not substantially improve the correspondence of ionization energies to deep KS levels.^114,115^ While this approach is not exact, it is at the very least valid under our conditions, where IPA calculations reproduce the full TDDFT results in the case of LDA and PBE functionals (see the SI). For HHG, these ionization potentials are crucial when analyzing spectra, because the cutoff scales linearly with the *I*_*p*_ with the established cutoff law:^9^
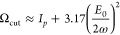
6where the relationship in eq 6 should hold for each MO separately, from the HOMO down to deeper states (contributions from several levels generally appear simultaneously in spectra). Equation 6 can be used to benchmark the validity level of each individual spectra, with deviations expected to be small even in the presence of multielectron effects and long-range interactions.^116−118^ We also note that eq 6 is slightly modified in molecular systems due to two-center (or multiple-center) effects.^119^ However, for our chosen laser parameters, this amounts to a cutoff shift of only ∼1 eV, below the energy of a single harmonic order. We further emphasize that, even in the absence of direct comparisons to experimental data, we can use eq 6 to point out clear and drastic deviations from well-established physical expectations. While this idea is somewhat precarious given that in general interactions it could alter eq 6, we believe this could be a starting point for a type of self-consistent benchmarking of theory against itself to ensure that expected physical behaviors are upheld (e.g., theory predicting reasonable energy scales for electronic kinetic energies and physical temporal dynamics). It is especially a good starting point in simple systems where interactions are relatively small.

Figure 2 presents HHG emission from randomly oriented N_2_ in conditions where the spectral minima is not present, since the driving laser power is lower. We choose to explore randomly oriented media for two main reasons: (i) These are the most typical experimental conditions, meaning that spectral features obtained here could serve as predictions for experiments to benchmark theory. (ii) By orientation averaging the HHG response, we partially eliminate minor artifacts due to the molecular symmetry, multicenter interferences, and additional noise. The orientation-averaged response also shows a reduced contribution from long trajectories and provides additional channels for decoherence. Overall, it allows to more easily single-out contributions coming from the level of theory and compare different molecules on equal settings.

**Figure 2:**
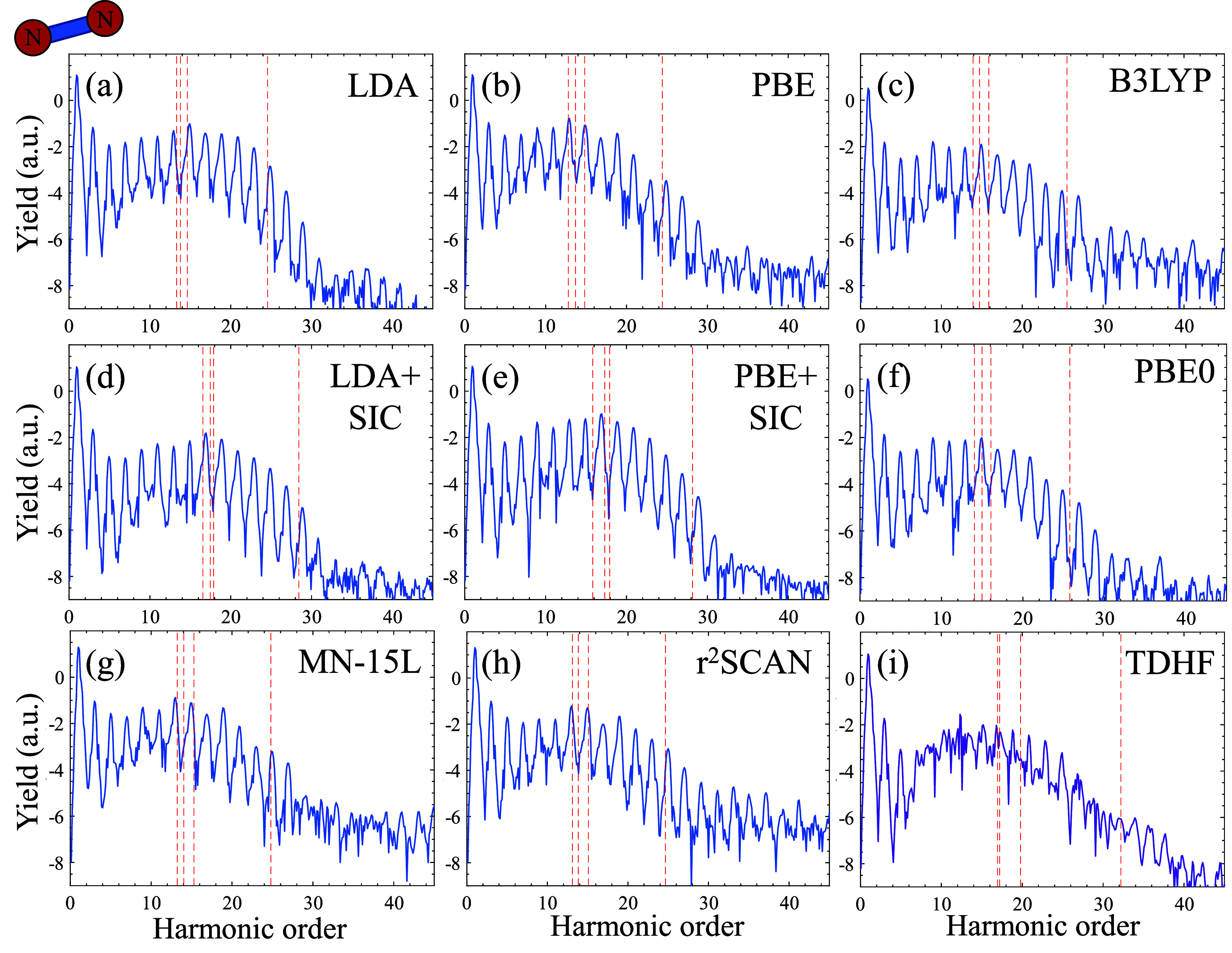
HHG spectra from N_2_ driven by 800 nm pulses at a power of 10^14^ W/cm^2^ with different levels of theory. Dashed red lines represent semiclassical HHG cutoff prediction from individual KS-MOs of N_2_, starting from the HOMO level (leftmost dashed line) to HOMO–4 (rightmost dashed line). Plot is given in logarithmic scale.

Let us analyze the results in Figure 2, while noting that cutoff positions can only be approximately numerically determined up to ±2 harmonic orders, but still discussing the main trends. The dashed red lines in all plots indicate the expected cutoff positions from the five topmost KS-MOs in N_2_ with their respective level of theory (corrected for two-center effects). The XC functional leading to the closest cutoff position is PBE with a SIC (PBE+SIC, overshooting the cutoff by only ∼0.7 eV) with LDA+SIC leading to very similar results. Both of these functionals correctly predict a sharp exponential decay region in the HHG spectra, following the cutoffs of the four topmost KS-MOs that have close *I*_*p*_ values (with the HOMO–4 level not making a substantial physical contribution—the highest-energy red dashed line in all spectra in Figure 2 denotes the SFA-expected cutoff from the HOMO–4 level, which does not correlate with any HHG features). B3LYP also leads to reasonable and similar-shaped spectra, but overshoots the expected cutoff by an additional four harmonic orders (∼6 eV). This by itself is not an issue, especially considering that the KS eigenvalues of deeper MOs are not supposed to match the ionization potentials anyway. However, B3LYP fails to reproduce a sharp exponential cutoff region, instead showing a weaker decay and noisy harmonic emission well beyond the cutoff (beyond 45 eV). Such phenomena are not expected from experiments performed in the last few decades in other systems and conditions, and generally one does not expect the physical interaction to produce such high-energy electrons. Even the presence of strong interactions should not change the energy scales by that much. PBE0 shows almost identical spectra to B3LYP, meaning that, in the case of hybrids, empirical or nonempirical functionals perform very similarly. For this system, note that TDHF also fails to obtain a proper cutoff position and produces noisy spectra (in accordance with previous work,^91^ which may indicate a more systematic failure). LDA and PBE functionals without a SIC leads to poorer results (missing the expected cutoff energy by ∼9 and 6 eV, respectively). Lastly, Figures 2g and 2h) show calculated HHG spectra with mGGA MN-15L and r^2^SCAN functionals, which, at first glance, provide a sharp exponential cutoff region and only miss the expected cutoff by ∼6 eV. In general, both functionals provide very similar spectra. However, similar to hybrids, mGGA produces noisy harmonics that are much too high in energy, and we will show below that they fail to produce proper time-frequency characteristics of the highly nonlinear response.

Besides benchmarking theory based on eq 6, we further explore the time-frequency characteristics of the emission by calculating a time-windowed Fourier transform (Gabor analysis):^120^
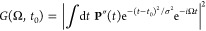
7where we use . Equation 7 resolves the contribution of electron trajectories to HHG because it allows one to extract the emission times of different harmonics, which corresponds to electronic recombination times in the semiclassical picture.

Figure 3 presents the corresponding Gabor transforms to the spectra in Figure 2. The expected physical behavior in this case is somewhat more difficult to pin down due to the multiorbital and multicenter nature of the N_2_ molecule, as well as due to the orientation averaging. Still, almost in every gas-phase HHG experiment (driven by a multicycle monochromatic linearly polarized laser), two main sets of trajectories are expected: a short and long trajectory, which coalesce at the cutoff and exhibit a bell-curve-like shape in the time-frequency domain.^40^ Typically, the long trajectories are suppressed. The semiclassical expectation is plotted as a dashed line for the HOMO orbital on top of all plots in Figure 3 for illustration purposes (note that it is not corrected for two-center effects, which are negligible). This behavior is largely reproduced by almost all levels of theory. In particular, we note that hybrid functionals yield qualitatively similar Gabor plots to PBE/LDA+SIC, even though they require substantially more numerical resources (∼15 times more costly). On the other hand, TDHF and mGGAs both fail in reproducing these temporal-spectral features. Specifically, TDHF leads to a temporal shift of ∼500 attoseconds in the HHG emission of the long trajectories (which are actually not observed in other levels of theory and typically should not prominently appear in spectra). mGGAs predict sharp chirpless features in the spectra (harmonics of 10–20) that do not make much physical sense. Similar features are also found in LDA and PBE without a SIC, but they are much less pronounced there and are not completely chirpless in those cases. mGGAs also show a temporal shift of ∼300 attoseconds in the low energy short trajectories that does not appear with other functionals.

**Figure 3:**
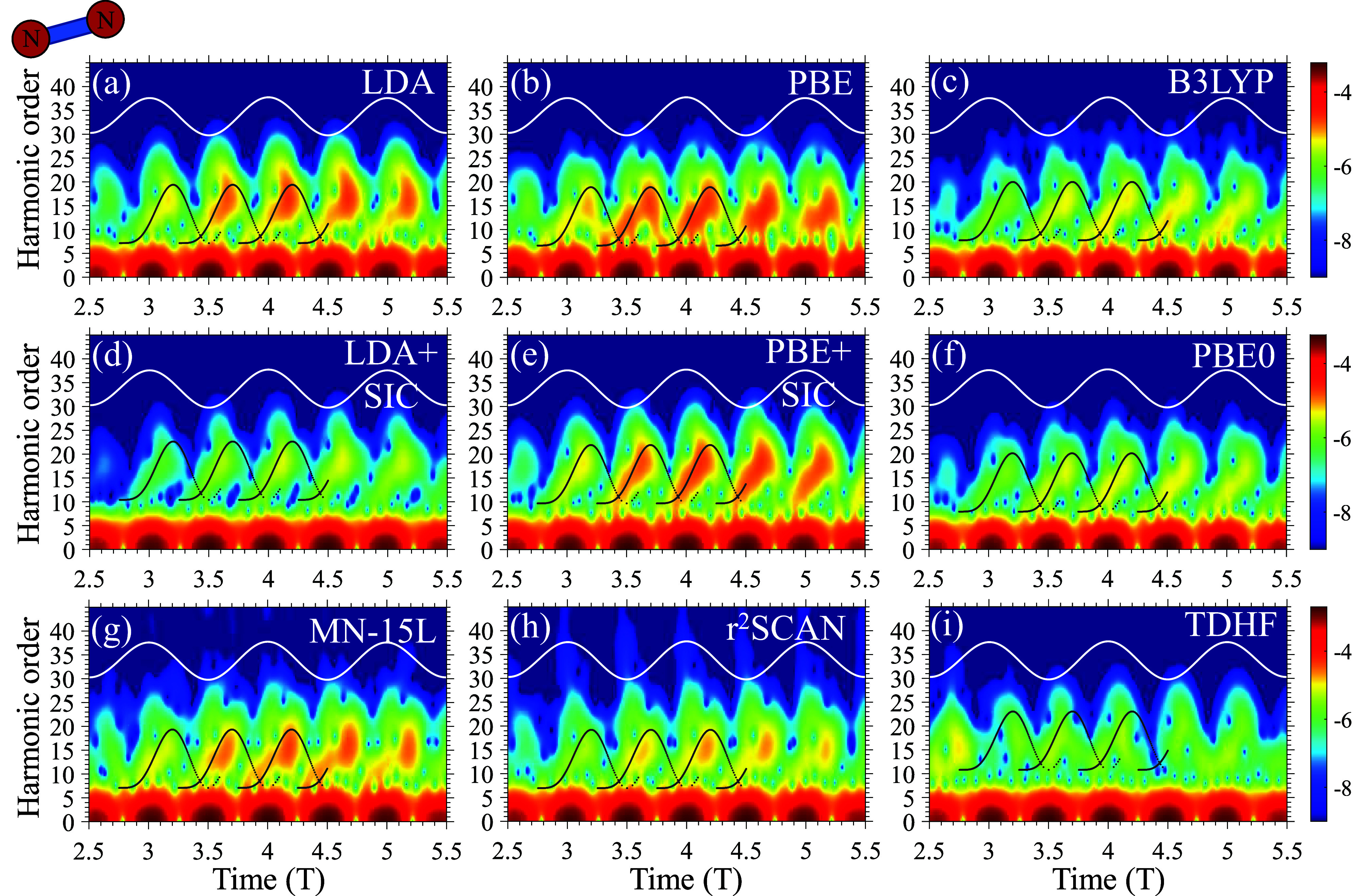
Gabor time-frequency plots corresponding to Figure 2. Black curves indicate semiclassical electron trajectories of the HOMO level (with short trajectories comprising the rising part of the bell-like curves and long trajectories comprising the downslope of these curves). White lines illustrate the driving laser pulse. Plot is given in logarithmic scale.

We next explore HHG from H_2_O gas, which has a lower-level symmetry (*C*_2v_). Gas-phase water HHG spectra have been previously measured in various settings (e.g. in refs (8) and (121)). In this case, due to the energy spacing of different molecular levels, several KS-MOs orbitals (from HOMO down to HOMO–3) can contribute to HHG. For driving pulses with a carrier wavelength of 800 nm, very similar conclusions can be drawn to the case of N_2_ in terms of the validity of different theory levels (see data in the SI). However, when exploring our numerical simulations we noticed that slightly tuning the wavelength to 900 nm allows testing additional signatures for physical soundness. Figure 4 shows the resulting HHG spectra. Contrarily to results at 800 nm driving (see the SI), here LDA, PBE, and mGGAs predict that the HOMO–3 level contribute to the emission of a second HHG plateau that is exponentially suppressed, suggesting that tunnel ionization rates from the HOMO–3 level are non-negligible even though it is much more tightly bound (∼7.5–10 eV deeper than the HOMO, depending on the level of theory). On the other hand, both TDHF and hybrids predict no such secondary plateau. In HF, this deviation can be understood as a result of the higher *I*_*p*_ value of the HOMO–3 level, compared to the PBE level (see the SI), suppressing the emission. As such, the absence of a second plateau in TDHF is not a failure of the dynamical theory per say, but rather a failure of HF in reproducing reasonable energy levels. For B3LYP and PBE0 on the other hand, the HOMO–3 is less tightly bound than in PBE, meaning that the absence of a second plateau arises from different electron dynamics under the hybrid functionals. This result shows the volatility of highly nonlinear and nonperturbative calculations to the level of theory and parameters of the Hamiltonian. How can we ascertain which functional then is correct in its prediction, if at all?

**Figure 4:**
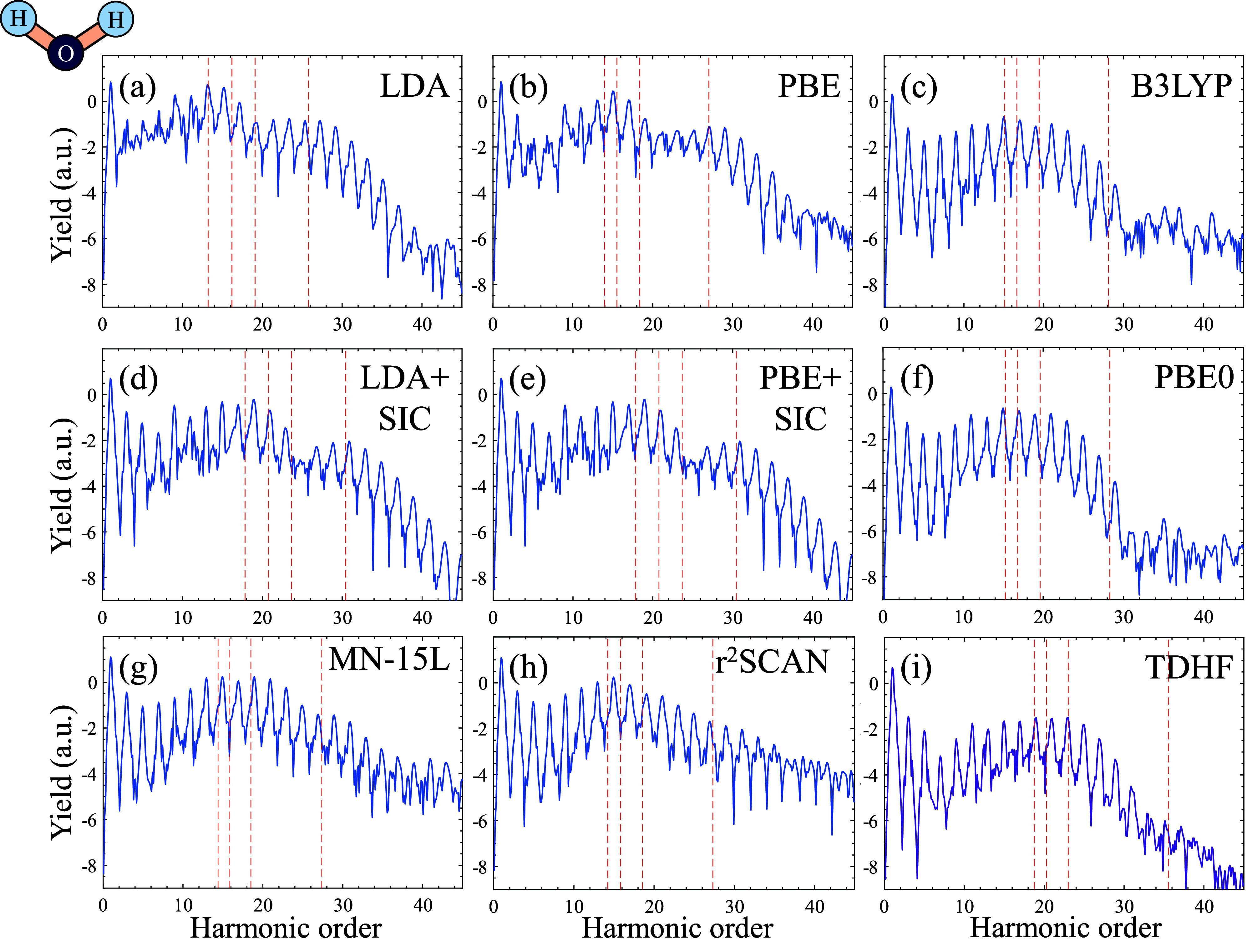
HHG spectra from H_2_O driven by 900 nm pulses at a power of 5 × 10^13^ W/cm^2^ with different levels of theory. Dashed red lines represent semiclassical HHG cutoff prediction from individual KS-MOs, starting from the HOMO level (leftmost dashed line) to HOMO–3 (rightmost dashed line). Plot is given in logarithmic scale.

Since we do not have reliable experimental data to verify or rule out the existence of a secondary plateau (although similar effects were in principle measured in other systems^122,123^), we compare the Gabor transforms obtained from two exemplary theory levels of PBE and B3LYP (see Figure 5; for additional Gabor plots for other theories, see the SI). The Gabor transforms are overlaid with the expected semiclassical electron trajectories for the dominant HOMO–1 and HOMO–3 levels. While it is difficult to draw a definitive conclusion, PBE better agrees with the expected trajectories, suggesting that the hybrid calculations exhibit nonphysical artifacts. This is especially prominent around the center of the main HHG plateau (harmonic orders of 15–20) where hybrids predict a strong negative chirp in the time-frequency emission, which is generally uncommon in gas-phase HHG. Due to hybrids typically overshooting expected cutoffs by ∼8 eV anyway, it is possible that a potential second plateau emission from the HOMO–3 level might be washed out by other KS-MO emissions. However, the fact that much too high energy and noisy harmonics are also observed up to 45 eV, together with the absence of a second plateau, suggests a possible more systematic failure.

**Figure 5:**
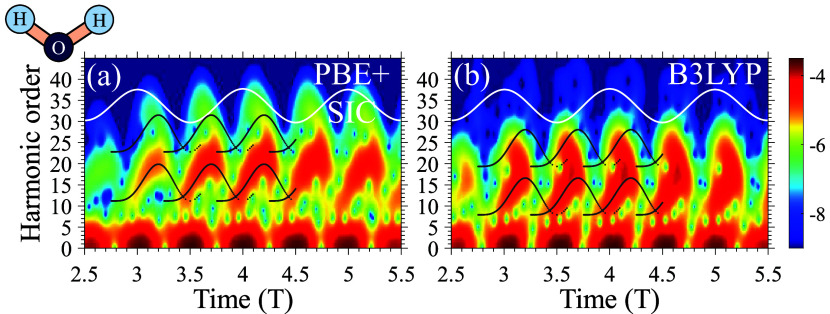
Gabor time-frequency plots corresponding to Figure 4 from (a) PBE+SIC, and (b) B3LYP. Black curves indicate expected semiclassical electron trajectories of the HOMO–1 (contributing to the first plateau) and HOMO–3 (contributing to the second plateau) levels. White lines illustrate the driving laser pulse. Plot is given in logarithmic scale.

To summarize, we performed extensive ab initio simulations of HHG driven by intense lasers in molecular gas-phase systems and standard laser regimes. We tested the effects of the level of theory, in particular, the choice of XC functional employed in adiabatic TDDFT, on the HHG response, and benchmarked it against standard well-established physical expectations such as the cutoff position, time-frequency characteristics, and HHG spectral structures. Our results allow drawing several critical conclusions: (i) When incorporating a self-interaction correction, the local density approximation (LDA) and generalized gradient approximation (GGA) produce nonlinear optical responses in good agreement with physical expectations. (ii) More elaborate functionals such as hybrids and mGGAs can, under certain conditions, fail and predict unphysical characteristics such as harmonic energies that are too high, overshooting cutoffs, wrong time-frequency characteristics, etc. Even if they do not fail, their application does not produce additional insight compared with simpler XC functionals, at least in our examined cases. (iii) TDHF can have systematic failure that does not allow reproducing expected spectral features. Therefore, our work at least partially validates the application of simpler XC approximation in the field, which was generally missing.

The main question arising from our study is what causes potential failures in more elaborate functionals in strong laser conditions. It is also not clear if the failure of hybrids is in some way connected to the failure of TDHF (both employing exact exchange) or not, or whether it is a general feature of hybrid and meta functionals or arises only in our chosen examples (it is possible one or more of the many other available hybrid/meta functionals avoids such issues). These questions should be a topic of future work and are beyond our scope. Still, we hypothesize several potential origins for such failures: (i) It could arise as a consequence of the failure of the adiabatic approximation in TDDFT, which might not hold under these conditions. It is possible that more-elaborate functionals have a higher susceptibility to such issues, resulting in reduced quality spectra and unphysical features. This is somewhat analogous to results obtained recently showing the numerical formulation of TDDFT can itself affect the extent of failure of the adiabatic approximation.^124,125^ (ii) Hybrid, meta, and higher level functionals might break more known properties of the exact functional (especially prominent for empirical functionals). It is possible that breaking these relations yields a yet unexplored failure for TDDFT. However, in our examined cases empirical and nonempirical functionals performed quite similarly, and SCAN (which upholds all known constraints of the exact functional) lead to similar failures. Thus, this hypothesis does not seem very likely. (iii) Specifically for mGGAs, the failures could be related to the choice of treating them either in the KS framework, solving the OEP equation (as done here) or within generalized KS equations. Furthermore, the solution of the OEP equation at the KLI level can lead to incorrect results.^126−129^ However, exemplary analysis that we have performed comparing the use of the KLI approximation to the generalized KS scheme does not seem to support this hypothesis (see further discussion in the SI), although it cannot be completely ruled out at this stage. Overall, understanding the origin of these failures (both in mGGA and hybrids) is an interesting open problem for future studies.

## Supporting Information Available

Details on the methodology used in numerical calculations presented in the main text, as well as complementary results in a variety of different physical settings (PDF)
