# Supplementary material for: Benchmarking Functionals for Strong-Field Light-Matter Interactions in Adiabatic Time-Dependent Density Functional Theory
Source: J Phys Chem Lett. 2024 Jul 8;15(28):7254–64. doi: 10.1021/acs.jpclett.4c01383 (PMC11261632; doi:10.1021/acs.jpclett.4c01383)
Supplement: Supplementary file 1 — jz4c01383_si_001.pdf [file jz4c01383_si_001.pdf]

# Supplementary Material: Benchmarking Functionals for Strong-Field Light-Matter Interactions in Adiabatic Time-Dependent Density Functional Theory

Ofer Neufeld[‡,1,], Nicolas Tancogne-Dejean[1], Angel Rubio[1,2,†]
[1]Max Planck Institute for the Structure and Dynamics of Matter and Center for Free-Electron Laser Science, Hamburg, 22761, Germany.
[2]Center for Computational Quantum Physics (CCQ), The Flatiron Institute, New York, NY, 10010, USA.

This supplementary material (SM) file contains technical details on the numerical calculations and methodology used in the main text, as well as some additional results.

- **S1: NUMERICAL DETAILS**

## 1. Ground state calculations

All calculations were performed using the octopus code[1–3]. The Kohn-Sham (KS) (or Hartree-Fock (HF)) equations were discretized on a Cartesian grid with a spherical shape of radius 35 Bohr and grid spacing of $\Delta x=\Delta y=\Delta z=0.38$ Bohr. When a self-interaction correction (SIC) was added, we included an average density form[4] that was employed fully self-consistently. The frozen core approximation was used for core states, which were treated with norm-conserving pseudopotentials[5]. Note that we employ only LDA and PBE pseudopotentials throughout, where PBE pseudopotentials are employed for all functionals (and HF) calculations apart from the LDA ones. This can also cause deviations in obtained eigenenergies, but is not expected to affect ultrafast dynamics in strong-fields. The KS (or HF) equations were solved to self-consistency with a tolerance $<10^{-8}$ Hartree. Molecular geometries were taken at their experimental and symmetric configurations. Hybrid functionals and HF calculations were treated with adiabatically compressed exchange[6]. In the main text mGGA calculations were performed within the KS framework, solving the OEP equations using the KLI approximation[7].

We also provide in Tables S1, S2 below the level alignments (KS or HF) eigenenergies for different occupied orbitals in each level of theory. The energies were used to determine semiclassical cutoff and trajectory plots overlaid on top of figures throughout the text. In HF, the order of the energy levels in $N_2$ is not consistent with the other approaches, as discussed in the main text (and in ref. 8). We checked that this is not due to the missing correlations, as pure exchange functionals like Slater and LDAx (not presented) show a consistent ordering of the energy level consistent with the functionals reported in Table S1.

**Table S1.** Molecular orbital (MO) eigenenergies in $N_2$ obtained for different theory levels in eV.

| $N_2$ MOs | LDA | LDA+SIC | PBE | PBE+SIC | B3LYP | PBE0 | MN-15L | $r^2$SCAN | HF | EXP. |
|---|---|---|---|---|---|---|---|---|---|---|
| HOMO | -11.090 | -16.139 | -10.313 | -15.013 | -12.035 | -12.246 | -10.986 | -10.785 | -16.749 | -15.581[9] |
| HOMO-1 | -11.832 | -17.560 | -11.702 | -17.310 | -13.288 | -13.680 | -12.236 | -11.925 | -16.749 | |
| HOMO-2 | -11.832 | -17.560 | -11.702 | -17.310 | -13.288 | -13.680 | -12.236 | -11.925 | -17.152 | |
| HOMO-3 | -13.147 | -18.202 | -13.442 | -18.247 | -15.053 | -15.370 | -14.184 | -13.854 | -21.140 | |
| HOMO-4 | -28.541 | -34.522 | -28.334 | -34.140 | -30.037 | -30.392 | -28.910 | -28.694 | -40.370 | |

**Table S2.** MO eigenenergies in $H_2O$ obtained for different theory levels in eV.

| $H_2O$ MOs | LDA | LDA+SIC | PBE | PBE+SIC | B3LYP | PBE0 | MN-15L | $r^2$SCAN | HF | EXP. |
|---|---|---|---|---|---|---|---|---|---|---|
| HOMO | -6.180 | -12.570 | -7.216 | -13.385 | -8.785 | -9.005 | -7.786 | -7.560 | -13.822 | -12.622[10] |
| HOMO-1 | -10.283 | -16.570 | -9.350 | -15.398 | -10.874 | -11.135 | -9.823 | -9.730 | -15.925 | |
| HOMO-2 | -14.285 | -20.584 | -13.278 | -19.238 | -14.720 | -14.993 | -13.449 | -13.507 | -19.668 | |
| HOMO-3 | -23.468 | -29.918 | -25.211 | -31.440 | -26.673 | -26.980 | -25.674 | -25.603 | -39.932 | |

## 2. Time-dependent calculations

For time-dependent DFT (TDDFT) calculations, the active KS orbitals were propagated with varying time steps and algorithms depending on the level of theory, which were all tested for convergence. We used in all cases the same Lanczos propagator with time steps of: 0.06 a.u. for meta-GGA, 0.15 a.u. for hybrids, 0.08 a.u. for HF, PBE, and LDA. In the cases of meta-GGA and hybrid functionals we employed a self-consistent propagation scheme[11]. We added a complex absorbing potential (CAP) with a width of 15 Bohr to all simulation boxes with a $\sin^2(r)$ shape to avoid boundary reflections[12]. The initial states were taken as the system's ground-states. HHG spectra were obtained as explained in the main text where the dipole acceleration was filtered with a super-gaussian window.

For mGGA propagation we employed the KLI approximation throughout the main text (as for the GS); however, this was done following exemplary comparison calculations to the generalized KS (GKS) formulation of TDDFT, which were shown to be in very good agreement, especially in the main plateau region (see Fig. S1(a) below). In all cases, we employ the U(1) gauge-invariant kinetic energy density[13–15]. In general, this good agreement between the KLI and GKS approaches for HHG suggests that errors coming from the KLI approximation are not the source of spurious behavior with mGGA functionals discussed in the main text. However, we cannot definitively rule those out as we have not verified every single test case and it remains unclear to which extent the KLI approximation could affect dynamics in such conditions.

Hybrid calculations were similarly performed, after comparison to the GKS formulation yielded essentially identical results (see Fig. S1(b) below).

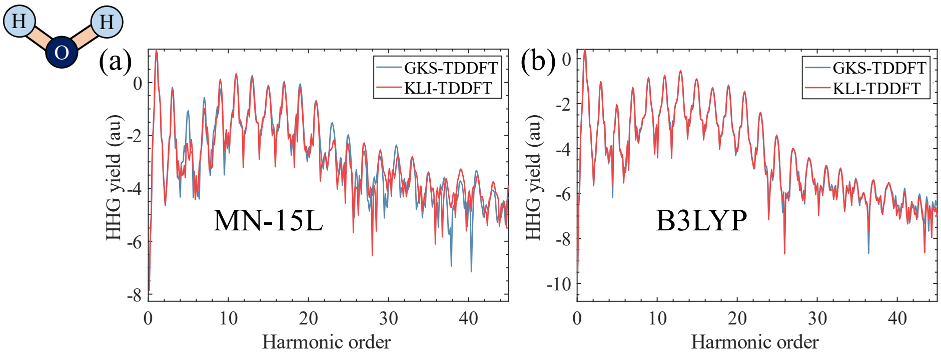


**Figure S1.** (a) HHG spectra calculated from $H_2O$ with MN-15L functional in similar conditions to those in the main text Fig. 4, but at 800nm driving, testing the application of the GKS formulation of TDDFT, or the application of the KLI approximation. The spectra are very similar, especially in the main plateau region of harmonic orders 10-20, validating that the failure of mGGA in that region appears in both implementations. (b) Same as (a) but for the hybrid B3LYP.

## 3. Orientation averaging

Orientation averaging was performed using trapezoidal weights with an angular grid spanned by Euler angles in the *z*-*y*-*z* convention. In $N_2$ we used an Euler angle spacing of $\pi/2$, and in $H_2O$ (lower-symmetry) a spacing of $\pi/4$. All angular grid spacings were tested for convergence. After utilizing the symmetries of linearly-polarized light in the dipole approximation, as well as $D_\infty$ symmetries in $N_2$, these grids lead to a total of 6, and 58 independent orientations in $H_2O$ and $N_2$, respectively (which are equivalent to full grids with 75, and 405 independent orientations, respectively).

## 4. Laser pulse

In time-dependent calculations for orientation averaged systems we employed the laser pulse described by eq. (3) in the main text, with the temporal envelope function taken as[16]:

$$f(t) = \left( sin\left(\pi \frac{t}{T_p}\right)\right)^{\left(\frac{\left|\pi\left(\frac{t}{T_p} - \frac{1}{2}\right)\right|}{w}\right)} \tag{1}$$

where $w$=0.75, $T_p$ is the duration of the laser pulse which was taken to be $T_p = 8T$ where $T = 2\pi/\omega$ is a single cycle of the fundamental carrier frequency (the full-width-half max is $4T$). This form is roughly equivalent to a super-gaussian pulse, but where the field starts and ends exactly at zero amplitude, which is numerically more convenient. For Fig. 1 in the main text that explored the spectral minima from aligned $N_2$ we used a trapezoidal laser envelope with three-optical cycle long rise and drop section, and total duration of ten optical cycles (to obtain the same conditions employed in the full CI study in ref. 17).

### 5. Semi-classical calculations

The semi-classical cutoff and trajectories were obtained in the standard approach: By solving classical Newtonian equations of motion for single-active electrons driven by a time-periodic laser field, and neglecting coulombic effects (in the strong-field approximation (SFA)) and multi-electron interactions. We assumed following the simple-man model an instantaneous ionization of electrons at the ionic center with electrons emerging from the tunnel exit with a zero velocity. The cutoff energies plotted on top of HHG spectra in the main text were corrected for multi-center effects by allowing for recombination on ions neighboring the tunnel ionization site (which in these conditions provides an additional ~1eV kinetic energy).

## ▪ S2: ADDITIONAL RESULTS

### 1. HHG and Time-frequency analysis of $H_2O$ driven at 800nm

We present the HHG and Gabor time-frequency analysis of $H_2O$ driven with 800nm laser pulses, complementary to Fig. 3,4 in the main text. Figure S2 shows the HHG spectra (top) and Gabor plots (bottom) overlaid with semi-classical trajectories of the HOMO-1 level for different levels of theory. The figure clearly indicates the failure of meta-GGA in obtaining proper time-frequency characteristics, instead showing a sharp chirp-less feature in the spectra, as well as noisy harmonics far beyond the cutoff in hybrids. This agrees with conclusions obtained from $N_2$ at similar driving wavelength.

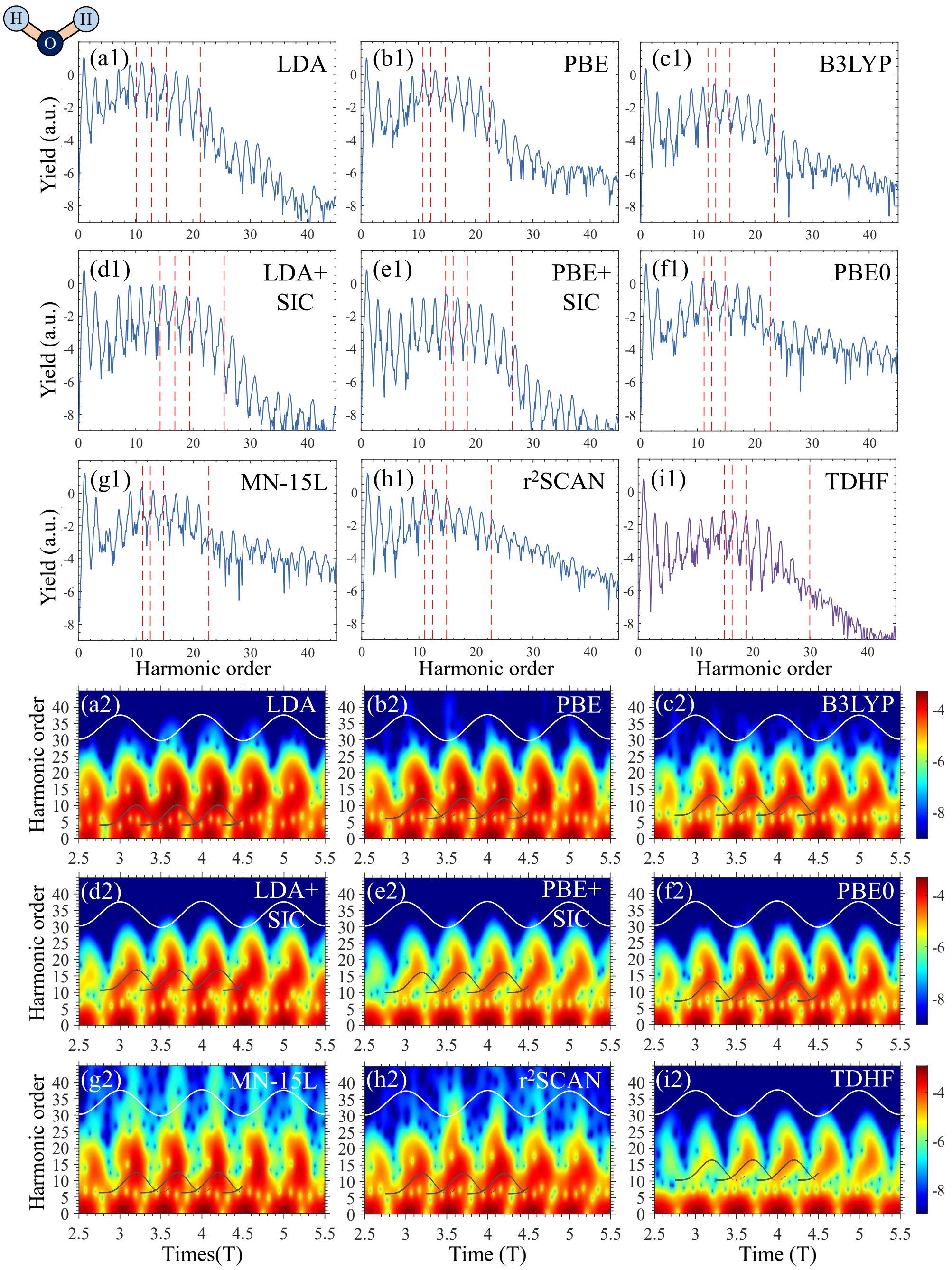


**Figure S2.** HHG (top, index 1) and Gabor plots (bottom, index 2) from randomly-oriented $H_2O$ driven at 800nm. Notations are similar to Fig. 2,3 in the main text but showing the semi-classical electron trajectories of the HOMO-1 level.

## 2. Time-frequency analysis of $H_2O$ driven at 900nm

We present the complete time-frequency analysis for all levels of theory in $H_2O$ driven at 900nm, complementing Fig. 5 in the main text. Figure S3 presents all Gabor plots overlaid with semi-classical electron trajectories for the HOMO-1 level.

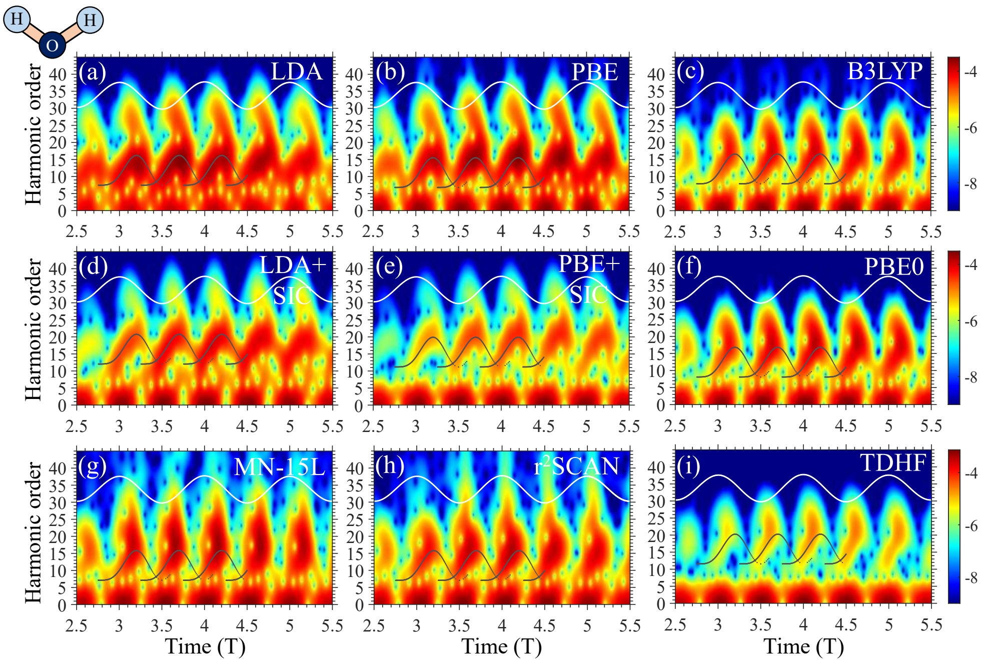


**Figure S3.** Corresponding Gabor plots to Fig. 4 in the main text (HHG in $H_2O$ driven at 900nm). Notations are similar to Fig. 5 in the main text but only showing the semi-classical electron trajectories from the HOMO-1 level.

## 3. IPA results for LDA

We present an exemplary comparison between HHG spectra calculated from $N_2$ with LDA with a SIC either employing or not the IPA (i.e. freezing the KS potential to its form at t=0 as discussed in the main text). As shown in Fig. S4, the differences after employing the IPA are rather minute, as typically the case in HHG calculations. This gives further validity to the semiclassical analysis in the main text, as well as to the notion of utilizing the vertical ionization energies of the different KS states. Especially important is that the HHG cutoff region is not affected by the IPA, further permitting the use of eq. (6) for attempting to benchmark theory.

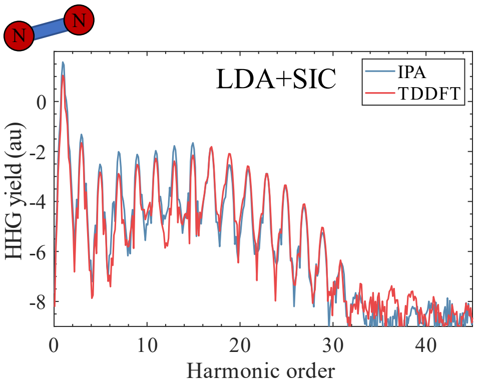


**Figure S4.** HHG spectra calculated from $N_2$ in similar conditions to those in the main text Fig. 2 (partially replicating Fig. 2(d)), but validating the application of the IPA in this laser and molecular regime.

## ▪ REFERENCES


(1) Castro, A.; Appel, H.; Oliveira, M.; Rozzi, C. A.; Andrade, X.; Lorenzen, F.; Marques, M. A. L.; Gross, E. K. U.; Rubio, A. Octopus: A Tool for the Application of Time-Dependent Density Functional Theory. *Phys. status solidi* **2006**, *243* (11), 2465–2488. https://doi.org/10.1002/pssb.200642067.

(2) Andrade, X.; Strubbe, D.; Giovannini, U. De; Larsen, H.; Oliveira, M. J. T.; Alberdi-rodriguez, J.; Varas, A.; Theophilou, I.; Helbig, N.; Verstraete, M. J.; Stella, L.; Nogueira, F.; Castro, A.; Marques, M. A. L.; Rubio, A. Real-Space Grids and the Octopus Code as Tools for the Development of New Simulation Approaches for Electronic Systems. *Phys. Chem. Chem. Phys.* **2015**, *17*, 31371–31396. https://doi.org/10.1039/C5CP00351B.

(3) Tancogne-Dejean, N.; Oliveira, M. J. T.; Andrade, X.; Appel, H.; Borca, C. H.; Le Breton, G.; Buchholz, F.; Castro, A.; Corni, S.; Correa, A. A.; De Giovannini, U.; Delgado, A.; Eich, F. G.; Flick, J.; Gil, G.; Gomez, A.; Helbig, N.;

Hübener, H.; Jestädt, R.; Jornet-Somoza, J.; Larsen, A. H.; Lebedeva, I. V; Lüders, M.; Marques, M. A. L.; Ohlmann, S. T.; Pipolo, S.; Rampp, M.; Rozzi, C. A.; Strubbe, D. A.; Sato, S. A.; Schäfer, C.; Theophilou, I.; Welden, A.; Rubio, A. Octopus, a Computational Framework for Exploring Light-Driven Phenomena and Quantum Dynamics in Extended and Finite Systems. *J. Chem. Phys.* **2020**, *152* (12), 124119. https://doi.org/10.1063/1.5142502.

(4) Legrand, C.; Suraud, E.; Reinhard, P.-G. Comparison of Self-Interaction-Corrections for Metal Clusters. *J. Phys. B At. Mol. Opt. Phys.* **2002**, *35* (4), 1115–1128. https://doi.org/10.1088/0953-4075/35/4/333.

(5) Hartwigsen, C.; Goedecker, S.; Hutter, J. Relativistic Separable Dual-Space Gaussian Pseudopotentials from H to Rn. *Phys. Rev. B* **1998**, *58* (7), 3641–3662. https://doi.org/10.1103/PhysRevB.58.3641.

(6) Lin, L. Adaptively Compressed Exchange Operator. *J. Chem. Theory Comput.* **2016**, *12* (5), 2242–2249. https://doi.org/10.1021/acs.jctc.6b00092.

(7) Krieger, J. B.; Li, Y.; Iafrate, G. J. Systematic Approximations to the Optimized Effective Potential: Application to Orbital-Density-Functional Theory. *Phys. Rev. A* **1992**, *46* (9), 5453–5458. https://doi.org/10.1103/PhysRevA.46.5453.

(8) Dauth, M.; Wiessner, M.; Feyer, V.; Schöll, A.; Puschnig, P.; Reinert, F.; Kümmel, S. Angle Resolved Photoemission from Organic Semiconductors: Orbital Imaging beyond the Molecular Orbital Interpretation. *New J. Phys.* **2014**, *16* (10), 103005. https://doi.org/10.1088/1367-2630/16/10/103005.

(9) Trickl, T.; Cromwell, E. F.; Lee, Y. T.; Kung, A. H. State-selective Ionization of Nitrogen in the X 2Σ+gv+=0 and V+=1 States by Two-color (1+ 1) Photon Excitation near Threshold. *J. Chem. Phys.* **1989**, *91* (10), 6006–6012. https://doi.org/10.1063/1.457417.

(10) Page, R. H.; Larkin, R. J.; Shen, Y. R.; Lee, Y. T. High-resolution Photoionization Spectrum of Water Molecules in a Supersonic Beam. *J. Chem. Phys.* **1988**, *88* (4), 2249–2263. https://doi.org/10.1063/1.454058.

(11) Castro, A.; Marques, M. A. L.; Rubio, A. Propagators for the Time-Dependent Kohn–Sham Equations. *J. Chem. Phys.* **2004**, *121* (8), 3425–3433. https://doi.org/10.1063/1.1774980.

(12) De Giovannini, U.; Larsen, A. H.; Rubio, A. Modeling Electron Dynamics Coupled to Continuum States in Finite Volumes with Absorbing Boundaries. *Eur. Phys. J. B* **2015**, *88* (3), 56. https://doi.org/10.1140/epjb/e2015-50808-0.

(13) Bates, J. E.; Furche, F. Harnessing the Meta-Generalized Gradient Approximation for Time-Dependent Density Functional Theory. *J. Chem. Phys.* **2012**, *137* (16), 164105. https://doi.org/10.1063/1.4759080.

(14) Richter, R.; Aschebrock, T.; Schelter, I.; Kümmel, S. Meta-Generalized Gradient Approximations in Time Dependent Generalized Kohn–Sham Theory: Importance of the Current Density Correction. *J. Chem. Phys.* **2023**, *159* (12), 124117. https://doi.org/10.1063/5.0167972.

(15) Aschebrock, T.; Lebeda, T.; Brütting, M.; Richter, R.; Schelter, I.; Kümmel, S. Exact Exchange-like Electric Response from a Meta-Generalized Gradient Approximation: A Semilocal Realization of Ultranonlocality. *J. Chem. Phys.* **2023**, *159* (23), 234107. https://doi.org/10.1063/5.0173776.

(16) Neufeld, O.; Cohen, O. Background-Free Measurement of Ring Currents by Symmetry-Breaking High-Harmonic Spectroscopy. *Phys. Rev. Lett.* **2019**, *123* (10), 103202. https://doi.org/10.1103/PhysRevLett.123.103202.

(17) Li, Y.; He, F.; Sato, T.; Ishikawa, K. L. Implementation of the Time-Dependent Complete-Active-Space Self-Consistent-Field Method for Diatomic Molecules. *J. Phys. Chem. A* **2024**, *128* (8), 1523–1532. https://doi.org/10.1021/acs.jpca.3c06799.
